# Impact of skin tone, environmental, and technical factors on thermal imaging

**DOI:** 10.1371/journal.pone.0325091

**Published:** 2025-09-10

**Authors:** Sharon Eve Sonenblum, Kathleen Jordan, Glory Tomi John, Andrew Chung, Miriam Asare-Baiden, Jordan Pelkmans, Judy Wawira Gichoya, Vicki Stover Hertzberg, Joyce C. Ho

**Affiliations:** 1 Nell Hodgson Woodruff School of Nursing, Emory University, Atlanta, Georgia, United States of America; 2 Department of Computer Science, Emory University, Atlanta, Georgia, United States of America; 3 Department of Radiology and Imaging Sciences, School of Medicine, Emory University, Atlanta, Georgia, United States of America; The Chinese University of Hong Kong, HONG KONG

## Abstract

**Background:**

Erythema, an early visual indicator of tissue damage preceding pressure injuries (PrIs), presents as redness in light skin tones but is harder to detect in dark skin tones. While thermography shows promise for early PrI detection, validation across different skin tones remains limited. Furthermore, most protocols and models have been developed under highly controlled conditions.

**Objective:**

To evaluate how environmental and technical factors (i.e., patient positioning, lighting, distance, camera type) and skin tone impact thermal imaging measurements and temperature change.

**Methods:**

This pre-post experimental study enrolled 35 healthy adults (30 with Monk Skin Tone Scale ≥6). Melanin Index was measured on the volar forearm using the SkinColorCatch^®^. After baseline imaging, a 15.5°C cooling stone was placed on one posterior superior iliac spine (PSIS) for 5 minutes. Thermal images were then collected with either the FLIR E8-XT or the FLIR ONE Pro camera under varied conditions: two lighting types (overhead room versus localized LED ring light), three postures (side-lying, side-lying with forward knee placement, and side-lying with rearward knee placement), and two camera-to-body distances (35 cm and 50 cm from the PSIS). The cooling/imaging procedure was repeated using the alternate camera, and data were analyzed using mixed-methods model.

**Results:**

Temperature change was effectively detected across all skin tones, with cooling resulting in a −3.7 ± 1.2°C difference between the region of interest (ROI) and control region. Camera type significantly affected measurements, with the ONE Pro recording 1.04°C less cooling than the E8-XT. Distance had minimal impact (0.11°C cooler at 50 cm vs 35 cm at baseline), with no significant difference when comparing ROI to control regions, while lighting and posture had no impact on measurements. Skin tone varied by cooling measurement, with higher melanin levels showing greater temperature changes. A 0.98°C difference was observed between the lightest and darkest skin tone groups.

**Discussion:**

Our findings confirm thermal imaging’s robustness across varied environments, with the minor distance effects mitigated through perpendicular measurements and relative temperature comparisons. Significant discrepancies between thermal cameras (>1°C) highlight that these technologies cannot be used interchangeably when establishing thresholds. While effective across all skin tones, the observed differences in cooling response suggest tailored thresholds may be necessary for darker skin tones. Future research should focus on clinical validation across diverse populations to enhance PrI detection accuracy.

## Introduction

Thermal imaging measures heat radiating from the body and is a tool with many potential clinical applications.

Of particular interest is the use of thermography for early detection of pressure injuries (PrIs), which has shown promise. A PrI is defined as “a localized injury to the skin and/or underlying tissue usually over a bony prominence or related to medical or other devices, as a result of pressure, or pressure in combination with shear” [1,2]. The underlying tissue damage may involve reduced blood flow (ischemia) or inflammatory responses, both of which alter local skin temperature, making thermal imaging a promising detection tool that can identify temperature variations indicating early tissue damage before visible changes occur [1,3]. Research demonstrates its potential for both early identification and reduction of PrI development rates [3–11].

Thermal imaging has also been used to detect acute appendicitis [12], deep vein thromboses [13], coccyx pain [14], myofascial trigger points [15], hypoperfusion in critically ill patients [16], and wound healing [17,18], among other applications. Despite widespread application, studies do not report the skin tone of participants and when available, participants with very dark skin tones were not studied.

This research gap is particularly concerning because erythema, an early visual indication of tissue damage that often precedes a PrI, presents differently across skin tones. While erythema typically appears as redness in persons with light skin tones, it often presents as hyperpigmentation in individuals with dark skin tones, making it more difficult to detect. This difficulty detecting skin color changes in persons with dark skin tones contributes to underlying health disparities related to PrIs, such as discovery at later stages and slower healing among Blacks, Hispanics, Native Americans, and Asians [19–21]. Without early detection, individuals with dark skin tones suffer disproportionately, enduring longer hospital stays, have more severe infections, reduced wellbeing, and in some cases, premature death [19,20,22–24]. Thus, it has been suggested that non-invasive bedside accessible technologies are needed to identify PrIs to reduce health disparities due to skin color [25–28]. In fact, thermography was highlighted in the 2019 International Pressure Injury Prevention and Treatment Clinical Practice Guideline as a priority for future research [1].

However, as mentioned previously, limited work has been done to validate the use of thermal imaging across different skin tones. Emissivity of human skin does not vary according to skin pigmentation, meaning that the thermal camera’s settings do not need to be changed according to the patient’s skin tone, a finding that is promising for the potential of thermography to be effective across diverse skin tones [29]. However, many other environmental, camera, and subject related factors may contribute to the accuracy of thermal measurements [30,31]. Furthermore, in a recent review on thermography’s accuracy in PrI detection, only one out of the eight included papers considered skin tone [3]. That study acknowledged the technology’s effectiveness on Fitzpatrick Skin Types I to III, but identified a need for improved approaches in darker skin tones [32]. A recent clinical trial of a thermography system in a long-term care setting did not report the demographics of the population nor the efficacy on the patients with dark skin, but the city in which the hospital was located included approximately 10% African American residents [11].

There have been several devices that, when used unvalidated in individuals with dark skin, have turned out to have inadequate performance resulting in real world consequences. For example, over-estimation of oxygenation with pulse-oximetry led to delays in treatment for African American patients [33,34], while infrared thermometers under-reported fevers in African Americans admitted to the hospital [35].

Furthermore, guidelines for using thermography dictate highly controlled conditions, specifying everything from the camera distance and room lighting to the floor surface and HVAC conditions [36,37]. Most studies designed to develop a protocol for detecting erythema similarly use highly controlled conditions [8,10]. Clinical environments are not nearly that controlled, resulting in challenges when implementing models developed in controlled environments into the real world. Therefore, the objective of this study was to evaluate how environmental and technical factors (i.e., patient positioning, lighting, distance, camera type) and skin tone impact thermal imaging measurements and temperature change.

## Materials and methods

This study used a pre-post experimental design with multiple instances of induced skin cooling on the lower backs of healthy adults.

### Participant population

This study enrolled 35 healthy adults between March 26^th^ and June 25^rd^ of 2024, 30 of whom had dark skin tones, defined as Monk Skin Tone (MST) Scale level 6 or greater when measured at the inner forearm, and 5 who had skin tones with MST level 5 or lower [38]. Participants had to be able to consent and were excluded if they did not speak English, were a member of a special population including women identifying as pregnant, individuals with a diagnosed communicable skin disease, individuals with a skin disease that might have been irritated by inducing erythema, or a bleeding disorder that made the individual prone to bruise more easily. This study was approved by Emory University’s Institutional Review Board (eIRB number 00005999).

### Instrumentation and measurements

#### Demographic data.

Participants completed an electronic REDCap survey containing questions about demographic data and information about height, weight, and smoking status.

#### Colorimetry.

The SkinColorCatch® (Delfin Technologies Ltd, Kuopio, Finland), a digital colorimeter, was used to describe the Melanin Index of the participant’s forearm. The SkinColorCatch® measures reflected light from the skin with a red, green, blue (RGB) color sensor. The SkinColorCatch® calculates a melanin index based on changes in the red-green light absorption, which are impacted by hemoglobin and melanin levels in the skin [39,40]. Skin tone at the inner forearm was classified using the modified Eumelanin Human Skin Colour Scale (Eumelanin Scale-Modified) [41,42], adapted from the scale initially described by Dadzie, et al. [43]. The melanin indices for the modified Eumelanin Human Skin Colour Scale were defined as Eumelanin low: < 25, Eumelanin intermediate low: 25 to <37.5, Eumelanin intermediate: 37.5 to <50, Eumelanin intermediate mid: 50 to <75, Eumelanin intermediate high: 75 to <100, and Eumelanin high: ≥ 100. Melanin index cutoff values were based on measurements from the ColorMeter DSM III from Cortex Technologies. Similarly, the measurements used in the Dadzie study included data collected on the DermaSpectrometer (Cortex Technologies), the ColorMeter DSM II (Cortex Technologies), and the Mexameter-MX18 (Courage+Khazaka Electronics, GmbH), which are related to but not identical to the Melanin Index measurements from the SkinColorCatch®, so a conversion equation was ascertained as described below.

The melanin index of 28 different skin tone swatches from across the PANTONE SkinTone^TM^ Guide (Pantone LLC, Carlstadt, NJ) were measured in triplicate with both the SkinColorCatch® and the ColorMeter DSM II. The average melanin values from each device were graphed and a logarithmic relationship between the two devices was identified (R^2^ = 0.9968). The logarithmic equation (Equation 1) was used to convert the SkinColorCatch® melanin values measured at the forearm to the ColorMeter DSM II scale and Eumelanin Scale-Modified categories, accordingly.


$$y=e^{\left(\frac{\left(x+623.18\right)}{334.81}\right)}$$

**Equation 1. The logarithmic equation used to convert the SkinColorCatch® melanin values measured at the forearm to the ColorMeter DSM II scale needed for determining the Eumelanin Scale-Modified categories. X represents the Melanin Index measured by the SkinColorCatch® and y represents the melanin value that is used to categorize the Eumelanin Scale-Modified categories.**

#### Thermal imaging.

The FLIR E8-XT and the FLIR ONE Pro (FLIR Systems, Inc., Wilsonville, OR) thermal cameras were used to collect optical and thermal images of the sacral region throughout the study. The FLIR E8-XT is an industrial camera with higher resolution and accuracy, while the ONE Pro is a consumer grade device with lower resolution and accuracy. The technical specifications of both cameras can be found in the Supplementary Materials Table 6 S1 File. The Multi-Spectral Dynamic Imaging (MSX) image setting was used, allowing the visual and thermal image to be seen in a single fusion image.

#### Data collection.

The participants’ posterior superior iliac spines (PSIS) were palpated while the participant was standing. A 2” circle was drawn on the skin around each PSIS using an eyeliner pencil with high color contrast to the participant’s skin tone. This circle was traced to ensure a solid and clear marking. The right PSIS was used for the cooling protocol.

**Fig 1:**
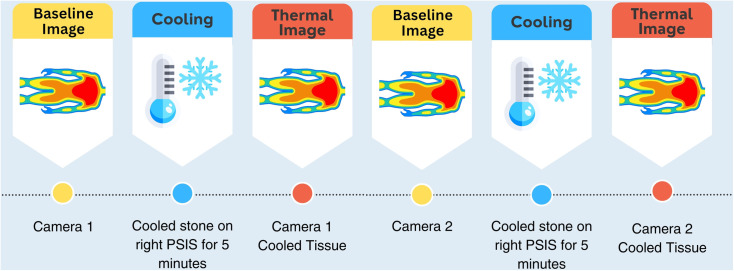
Study protocol includes two cooling interventions – once for each test camera.

**Fig 2:**
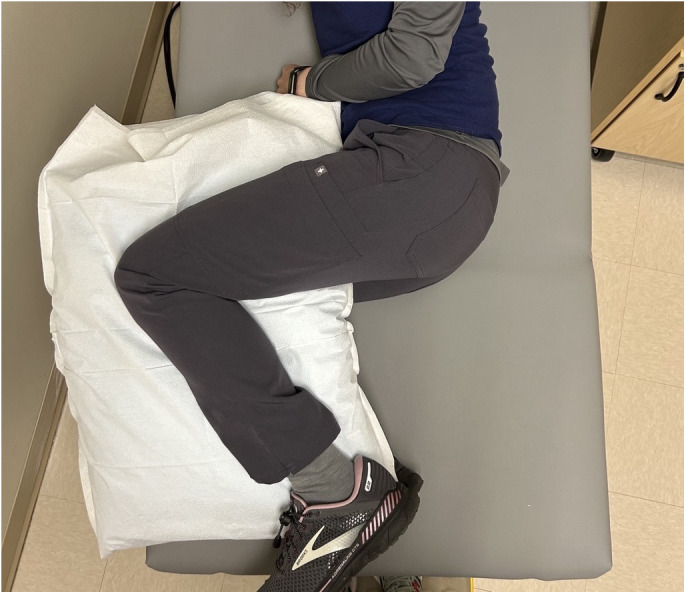
Example of knees stacked posture for data collection.

The overall flow of the study was conducted according to Fig 1. This generally occurred after the participant had approximately 15 minutes to acclimate to the room while the consent process and demographic survey were completed. While the participant was on their side, a series of baseline images were collected with the first, randomly selected thermal camera. These images were taken under different combinations of two lighting conditions (ambient/room lighting on and a ring LED light with room lights off), two camera distances (35 and 50 cm), and 3 postures (Table 1, and knees stacked example in Fig 2) for a total of 12 unique images. The order of the combinations was randomized for each participant. Next, the patient was asked to lay prone, while a stone that had been cooled in a 15.5° C (60° F) water bath was placed on their right PSIS for 5 minutes. The participant then returned to lying on their side to repeat the same series of 12 imaging conditions with the first camera to capture post-cooling measurements. This ended the first cooling protocol with 24 pre-post paired images, 12 baseline and 12 cooled.

**Table 1:** Conditions for thermography images will consider the variations experienced in a clinical environment.

Lighting	Distance from PSIS	Patient Position
Ambient (Room) Lighting	35 cm	Side-lying with legs on top of one another and pillow between knees (knees stacked)
Room lights off, bright LED ring light on	50 cm ^19^	Side lying with forward placement of top knee, creating an increase in flexion of the top hip (knee forward)
		Side-lying with rearward placement top knee, creating a decrease in flexion of top hip (knee behind)

After the first cooling protocol, the region of interest (ROI) was given approximately 15 minutes to return to baseline temperature before the cooling protocol was then repeated on the right PSIS. This time images were collected with the second thermal camera. The order of the 12-image series (i.e., the randomized sequence of the combinations of lighting, distance, and patient positioning conditions) was kept the same as with the previous thermal camera. This completed the study visit with a total of 48 pre-post paired cooled images.

### Data processing

The corner points on the identification card and/or sticker visible in the image were mapped between the optical and thermal images with an affine transformation, aligning the two images.

**Fig 3:**
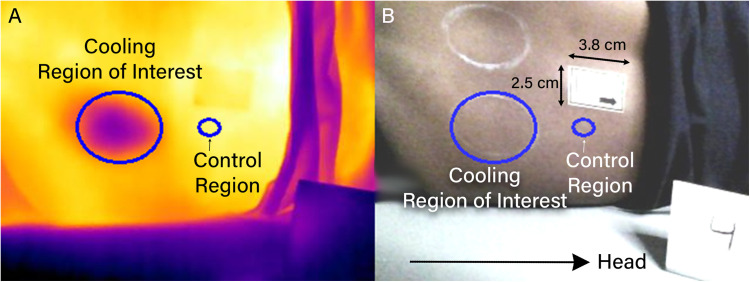
Regions of interest for the cooling protocol shown on (A) thermal and (B) optical images after alignment. The cooling ROI (large ellipse) is positioned over the posterior superior iliac spine (PSIS); the control ROI (small ellipse) is located superior to the PSIS.

To process the cooling images, two ROIs were selected (Fig 3). An elliptical region of interest was selected using a custom Python script that matched the outline of the circle drawn on the PSIS. We defined the ROI by placing a minimum of five points along the border of the circle and used these points to generate a best-fit ellipse that encompassed the cooling region.

A standardized control region was automatically created as an ellipse scaled proportionally to the cooling ROI, with axes measuring 1/4th the length of the long and short axes of the cooling ROI. This scaling ensured the control region captured a consistent proportion of tissue across participants regardless of anatomical variations. The control region was positioned superior along the x-axis of the image from the center of the original cooling ROI, offset by the width of the ellipse plus the radius of the long axis of the original cooling ROI. On some occasions, this placed the control region overlapping with the sticker or clothing, in which case it was manually relocated to the nearest appropriate location consistent with the originally defined parameters while avoiding any overlap. This standardized approach balanced the need for adequate tissue sampling against the sensitivity of single-point measurements and the challenge of using full-sized control ROIs (which would face inadequate control region availability).

### Data analysis

For each participant and experimental condition, we calculated within-subject averages and used these subject-level averaged data to perform two statistical analyses: (1) one-sample t-tests (∆ = 0) on temperature differences between baseline and cooling periods within each region (i.e., Cooling and Control ROI) to determine if cooling produced significant changes, and (2) paired t-tests comparing temperatures between cooling and control ROI at individual timepoints (baseline or cooling). Cohen’s d was calculated for all tests to assess effect size, with values of 0.2, 0.5, and 0.8 representing small, medium, and large effects, respectively.

Linear mixed-effects models were used to evaluate the effect of camera type on temperature measurements. A separate linear mixed-effects model, using only data collected with the FLIR E8-XT camera, was conducted to evaluate the effects of distance, participant posture, lighting, and skin tone on median temperature differences between the ROI compared to the control region. Skin tone was analyzed as a continuous variable using melanin index measurements obtained from the forearm using the SkinColorCatch. All models included subject ID as a random effect (random intercepts) and the respective experimental factors as fixed effects, with either absolute temperature or temperature difference serving as the dependent variable. For skin tone, we examined both its main effect and its interaction with cooling condition. Models were fit using the lme4 package in R (R version 4.5.0 (2025-04-11)), with p-values calculated using Satterthwaite’s method for denominator degrees of freedom.

## Results

### Participants

This study included 35 participants who varied in age, ranging from 18 to 73 years old (Table 2). An initial sample size of 30 participants was determined based on feasibility considerations and available resources. We recruited 30 participants who had an MST level of 6 or greater, and, following initial data review, we added 5 participants who had an MST level of 5 or lighter to ensure adequate representation across the skin tone spectrum. Study participants included more women, and were evenly dispersed between those of normal BMI, overweight and obese. Due to our enrollment strategy of including 30 participants who had a MST level of 6 or greater, most participants were in the Intermediate Mid Modified Eumelanin Skin Tone Category [41].

**Table 2:** Participant characteristics.

Characteristic	N = 35^1^
**Age (Years)**	39.71 ± 16.45
**Sex**	
Female	24 (69%)
Male	11 (31%)
**BMI (kg/m**^**2**^)	29.41 ± 6.83
**BMI Category**	
Normal	13 (37%)
Obese	14 (40%)
Overweight	8 (23%)
**Race**	
Asian	5 (14%)
Black or African American	23 (66%)
White	3 (9%)
More than one race	4 (11%)
**Ethnicity**	
Hispanic or Latino	2 (5.7%)
Not Hispanic or Latino	33 (94%)
**Smoking Status**	
Former smoker	6 (17%)
Has never smoked	29 (83%)
**Modified Eumelanin Skin Tone Category**	
Intermediate Low	4 (11%)
Intermediate	6 (17%)
Intermediate Mid	22 (63%)
Intermediate High	3 (8.6%)
**Melanin Index**	55.94 ± 13.54
**Monk Skin Tone Group**	
2	3 (8.6%)
4	1 (2.9%)
5	1 (2.9%)
6	7 (20%)
7	22 (63%)
8	1 (2.9%)

### Temperature change after cooling

Temperature in the ROI was 30.8°C ± 0.9°C prior to cooling and use of the cooling stone resulted in significant cooling of 3.2° C ± 0.6° C (t = −31.31, df = 34, p < 0.001, d = −5.29), representing a large effect size (Table 3). During cooling, the temperature difference between regions increased substantially, with the Cooling ROI being −3.7°C ± 0.8°C cooler than the Control Region (t = −28.17, df = 34, p < 0.001, d = −3.55), demonstrating a large effect. While there was a small but significant baseline difference between regions (−0.3°C ± 0.5°C, t = −3.33, df = 34, p = 0.002, d = −0.16), this represented a negligible effect. The control region showed minimal temperature change following cooling (0.2°C ± 0.5°C warmer than baseline, t = 1.97, df = 34, p = 0.057, d = 0.33)).

**Table 3:** Temperature responses during baseline and cooling in both regions of interest.

Median Temperature in Region	Baseline N = 35^1^	Cooled N = 35^1^	Cooled – Baseline N = 35^1^
Cooling Region of Interest (deg C)	30.8 ± 0.9	27.5 ± 1.1	−3.2 ± 0.6
Control Region	31.1 ± 0.8	31.2 ± 0.9	0.2 ± 0.5
Cooling Region of Interest – Control Region	−0.3 ± 0.5	−3.7 ± 0.8	

^1^Mean ± SD.

### Differences across environmental and technical factors

**Fig 4:**
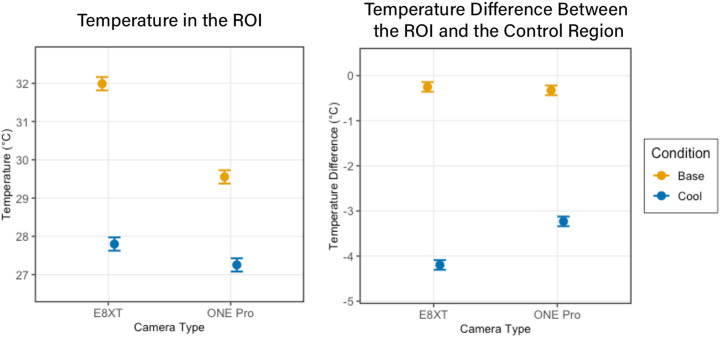
There was a significant impact of cooling and camera on the absolute temperature in the ROI (Left), whereas the temperature difference between the ROI and Control Region showed camera-dependent variation only in the cooling condition (Right).

A linear mixed-effects model revealed significant differences between cameras and their measurement of temperature changes (Fig 4). Under baseline conditions, measurements from the ONE Pro camera were 2.43°C lower than those from the FLIR E8-XT (p < 0.001). With the FLIR E8-XT camera, cooling reduced temperatures from baseline by 4.19°C (p < 0.001). However, a significant interaction between cooling and camera type (β = 1.89, p < 0.001) indicated that the difference in the measured temperature change between cameras was 1.89°C smaller when measured with the ONE Pro camera. When analyzing differences between ROI and control regions, which is more clinically relevant, there was no significant difference between cameras at baseline (β = −0.08°C, p = 0.113). After cooling, the ROI temperature decreased 3.95°C more than the control region when measured with the FLIR E8-XT (p < 0.001). A significant interaction term (β = 1.04°C, p < 0.001) indicated that this difference was 1.04°C smaller when measured with the ONE Pro camera. However, both devices still observed significant temperature changes across participants.

Further analysis was all run using only data collected on the E8-XT camera. Distance had small but variable effects on temperature measurements. At 50 cm, ROI temperatures were 0.11°C cooler than at 35 cm during baseline, though this difference was smaller during cooling (interaction β = 0.20°C, p = 0.045). Control regions showed a similar but non-significant trend to be cooler at 50 cm (β = −0.07°C, p = 0.177). When analyzing the more clinically relevant metric, the difference between ROI and control regions, measurements at 50 cm were not significantly different from those at 35 cm (β = 0.08°C, p = 0.122).

Linear mixed effects models confirmed a significant temperature reduction during cooling compared to baseline. However, neither patient positioning nor lighting conditions significantly affected the measurement of temperature change, whether assessed as absolute temperature in the cooling region or as the temperature difference between cooling and control regions. A combined linear mixed-effects model incorporating all factors yielded consistent results (Supplementary Table 5 in S1 File).

### Differences across skin tone

**Fig 5:**
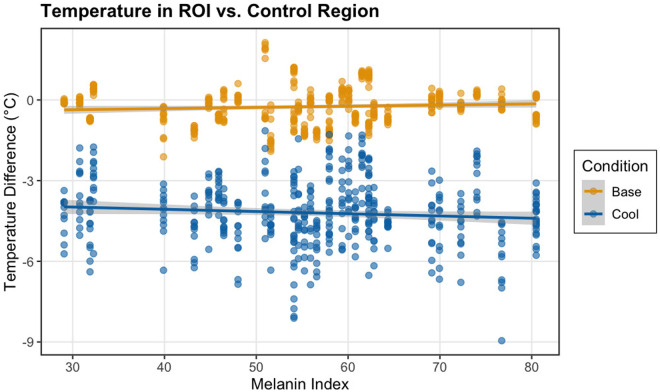
Measured temperature difference between the ROI and control region at baseline (yellow) and after cooling (blue) varies according to melanin index. Linear regression lines show the relationships between melanin index and temperature differences for each condition, with 95% confidence intervals indicated by gray shaded regions.

Linear mixed effects modeling revealed a significant interaction between melanin and cooling (p = 0.001), indicating that the thermal camera’s measurement of the temperature change varied with skin tone (Fig 5). While cooling produced an overall temperature reduction in the difference between the ROI and the control region (estimate = −3.24°C, p < 0.001), this difference was slightly increased in participants with higher melanin levels, as evidenced by the negative interaction term (estimate = −0.013°C per color unit, p = 0.001).

Because different skin tone categories are separated by 25 units, this would represent a measured difference of 0.325°C per skin tone category, or a difference of 0.975°C between the Intermediate Low and the Intermediate High skin tone categories.

## Discussion

### The impact of environmental and technical factors on thermal measurements and temperature change

Thermal imaging has been tested in highly controlled conditions and implemented within clinical bundles, but the impact of environmental conditions and skin tone have not been reported outside of manufactures’ 510(k) reporting [44]. The present study showed that the FLIR E8-XT thermal camera was not sensitive to lighting or posture. Absolute temperature measurements varied with distance, but the temperature difference between the ROI and control regions remained constant across distances. However, maintaining consistent measurement distances remains important for precise temperature assessment. From a clinical perspective, choosing a control region at a similar distance to the device, which is feasible if the skin surface is perpendicular to the thermal camera, should mitigate the impact of the distance effect. Additionally, varying distances from the camera within a single image, as would occur if the image is not taken perpendicular to the skin surface or is taken over a highly contoured area, could create more significant artifacts and therefore presents a greater concern for clinical interpretation.

In this study, we tested two camera models with substantially different specifications and costs. Our results revealed that these cameras reported temperature changes after cooling with different sensitivities, with discrepancies exceeding 1°C. This finding suggests that equivalency cannot be assumed across all thermographic imaging products. The more sensitive camera, the FLIR E8-XT, was an industrial-grade device with superior accuracy and resolution compared to the consumer-grade FLIR ONE Pro. This performance difference reflects the substantial gap in their intended applications and price points, suggesting that camera specifications may be an important consideration for clinical applications. Our results align with what might be expected given the technological diversity documented by Baron et al., who identified six different technologies implemented across eight studies [3]. Among the three devices with available information, measurement precision varied considerably (±2% to ±10%), as did temperature ranges (−20°C to 250°C, −10°C to 140°C, and −20°C to 400°C), while no specifications were available for the remaining three devices. This technological heterogeneity may contribute to the wide variety of temperature thresholds used and identified in the literature to determine tissue damage, ranging anywhere from 0.1°C [8,45], 1°C [46], 1.2°C [4], 1.5°C [47], and 2.2°C [32]. Although this list is not exhaustive, it highlights significant gaps in our understanding and the lack of standardization in accepted evidence. Based on these findings, researchers and clinicians should exercise caution when generalizing results and recommended thresholds across different thermographic devices without first considering their technical specifications.

### The impact of skin tone on thermal measurements and temperature change

Variations in temperature were observed across different skin tones after cooling, with participants with higher melanin content measuring colder after the same cooling protocol. This pattern resembles previous findings with infrared thermometers, where lower temperature readings in African-American patients led to the under detection of fevers, though the magnitude of difference was considerably different between studies [35]. Whether this variation stems from actual physiological differences in thermodynamic response to our intervention or from measurement artifacts related to thermal imaging remains undetermined. Nevertheless, two key conclusions emerged: First, thermal imaging successfully detected temperature changes across all skin tones, confirming its utility for detecting cooling in individuals with darker skin. This was anticipated based on the constant emissivity across skin pigmentation [29]. Second, our findings emphasize the necessity for rigorous clinical validation across diverse skin pigmentations. Since our research also aims to detect temperature changes due to pathological conditions such as inflammation or ischemia, thermal responses to these conditions may present differently across skin tones—whether due to physiological differences in vascular response or measurement factors. Regardless of the underlying mechanism, developing melanin-specific detection thresholds may be necessary to ensure accurate assessment across varied skin tones.

## Conclusion

In conclusion, this study provides valuable insights into the reliability and limitations of thermal imaging across diverse skin tones. Our findings demonstrate that while distance had a small but measurable effect on absolute temperature measurements, this can be effectively mitigated through perpendicular imaging and selecting control regions at consistent distances. The observed discrepancies exceeding 1°C between different thermal cameras indicate that these technologies cannot be used interchangeably, particularly when establishing detection and healing thresholds. Most notably, our discovery that participants with higher melanin content exhibited lower temperature readings following identical cooling protocols suggests that skin pigmentation affects thermal imaging assessment, although the underlying mechanism is unclear. These results emphasize the potential need for melanin-specific detection thresholds to ensure equitable diagnostic accuracy across diverse populations. Future research should focus on developing standardized protocols that account for both technological variations and skin tone diversity to improve the clinical utility of thermal imaging.

## Supporting information

S1 File Supplement Table. (PDF)

## References

[1] European Pressure Ulcer Advisory Panel National Pressure Injury Advisory Panel and Pan Pacific Pressure Injury Alliance. Prevention and treatment of pressure ulcers/injuries: clinical practice guideline. The International Guideline. EPUAP/NPIAP/PPPIA; 2019.

[2] Edsberg LE, Black JM, Goldberg M, McNichol L, Moore L, Sieggreen M. Revised national pressure ulcer advisory panel pressure injury staging system: revised pressure injury staging system. J Wound Ostomy Continence Nurs. 2016;43(6):585–97. doi: 10.1097/WON.0000000000000281 27749790 PMC5098472

[3] Baron MV, Hernandes Martins PR, Brandenburg C, Koepp J, Reinheimer IC, Dos Santos AC. Accuracy of thermographic imaging in the early detection of pressure injury: a systematic review. Adv Skin Wound Care. 2023;36(3):158–67. 36812081 10.1097/01.ASW.0000912000.25892.3f

[4] Farid KJ, Winkelman C, Rizkala A, Jones K. Using temperature of pressure-related intact discolored areas of skin to detect deep tissue injury: an observational, retrospective, correlational study. Ostomy Wound Manage. 2012;58(8):20–31. 22879313

[5] Cox J, Kaes L, Martinez M, Moles D. A Prospective, observational study to assess the use of thermography to predict progression of discolored intact skin to necrosis among patients in skilled nursing facilities. Ostomy Wound Manage. 2016;62(10):14–33. 27768578

[6] Bennett SL, Goubran R, Knoefel F. Long term monitoring of a pressure ulcer risk patient using thermal images. Annu Int Conf IEEE Eng Med Biol Soc. 2017;2017:1461–4. doi: 10.1109/EMBC.2017.8037110 29060154

[7] Mayrovitz HN, Spagna PE, Taylor MC. Sacral skin temperature assessed by thermal imaging: role of patient vascular attributes. J Wound Ostomy Continence Nurs. 2018;45(1):17–21. doi: 10.1097/WON.0000000000000392 29300285

[8] Cai F, Jiang X, Hou X, Wang D, Wang Y, Deng H, et al. Application of infrared thermography in the early warning of pressure injury: A prospective observational study. J Clin Nurs. 2021;30(3–4):559–71. doi: 10.1111/jocn.15576 33258199

[9] Wang Y, Jiang X, Yu K, Shi F, Qin L, Zhou H, et al. Infrared thermal images classification for pressure injury prevention incorporating the convolutional neural networks. IEEE Access. 2021;9:15181–90. doi: 10.1109/access.2021.3051095

[10] Pandey B, Joshi D, Arora AS, Upadhyay N, Chhabra HS. A deep learning approach for automated detection and segmentation of pressure ulcers using infrared-based thermal imaging. IEEE Sensors J. 2022;22(15):14762–8. doi: 10.1109/jsen.2022.3184105

[11] Holster M. Driving outcomes and improving documentation with long-wave infrared thermography in a long-term acute care hospital. Adv Skin Wound Care. 2023;36(4):189–93. 36790265 10.1097/01.ASW.0000912676.73372.a8

[12] Aydemir U, Sarıgoz T, Ertan T, Topuz Ö. Role of digital infrared thermal imaging in diagnosis of acute appendicitis. Ulus Travma Acil Cerrahi Derg. 2021;27(6):647–53. doi: 10.14744/tjtes.2020.80843 34710229

[13] Deng F, Tang Q, Zeng G, Wu H, Zhang N, Zhong N. Effectiveness of digital infrared thermal imaging in detecting lower extremity deep venous thrombosis. Med Phys. 2015;42(5):2242–8. doi: 10.1118/1.4907969 25979018

[14] Wu C-L, Yu K-L, Chuang H-Y, Huang M-H, Chen T-W, Chen C-H. The application of infrared thermography in the assessment of patients with coccygodynia before and after manual therapy combined with diathermy. J Manipulative Physiol Ther. 2009;32(4):287–93. doi: 10.1016/j.jmpt.2009.03.002 19447265

[15] Girasol CE, Dibai-Filho AV, de Oliveira AK, de Jesus Guirro RR. Correlation between skin temperature over myofascial trigger points in the upper trapezius muscle and range of motion, electromyographic activity, and pain in chronic neck pain patients. J Manipulative Physiol Ther. 2018;41(4):350–7. doi: 10.1016/j.jmpt.2017.10.009 29631764

[16] Luo JC, Zhang JD, Zhao QY, Wang H, Tu GW, Luo MH. Infrared thermography-based body-surface thermal inhomogeneity monitoring to assess the severity of hypoperfusion in critically ill patients. Shock. 2022;58(5):366–73. 36155398 10.1097/SHK.0000000000001998

[17] Yh L, Yc C, Ks C, Pj Y, Jl W, Ny K. Higher periwound temperature associated with wound healing of pressure ulcers detected by infrared thermography. J Clin Med. 2021;10(13). 10.3390/jcm10132883 PMC8269037 34209633

[18] Iruela Sánchez M, García-Sierra R, Medrano-Jiménez R, Bonachela-Mompart D, Maella-Rius N, Soria-Martín E, et al. Use of infrared thermometry to observe temperature variation associated with the healing process in wounds and ulcers: TIHUAP cohort study protocol. Healthcare (Basel). 2023;11(12):1750. doi: 10.3390/healthcare11121750 37372868 PMC10298734

[19] Bliss DZ, Gurvich O, Savik K, Eberly LE, Harms S, Mueller C, et al. Racial and ethnic disparities in the healing of pressure ulcers present at nursing home admission. Arch Gerontol Geriatr. 2017;72:187–94. doi: 10.1016/j.archger.2017.06.009 28697432 PMC5586547

[20] Gunowa N, Hutchinson M, Brooke J, Jackson D. Pressure injuries in people with darker skin tones: a literature review. J Clin Nurs. 2018;27(17–18):3266–75. 28887872 10.1111/jocn.14062

[21] Black J, Cox J, Capasso V, Bliss DZ, Delmore B, Iyer V, et al. Current perspectives on pressure injuries in persons with dark skin tones from the National Pressure Injury Advisory Panel. Adv Skin Wound Care. 2023;36(9):470–80. 37590446 10.1097/ASW.0000000000000032

[22] Saladin LK, Krause JS. Pressure ulcer prevalence and barriers to treatment after spinal cord injury: comparisons of four groups based on race-ethnicity. NeuroRehabilitation. 2009;24(1):57–66. doi: 10.3233/NRE-2009-0454 19208958

[23] Bliss DZ, Gurvich O, Savik K, Eberly LE, Harms S, Mueller C, et al. Are there racial-ethnic disparities in time to pressure ulcer development and pressure ulcer treatment in older adults after nursing home admission? J Aging Health. 2015;27(4):571–93. doi: 10.1177/0898264314553895 25260648 PMC5361740

[24] Bates-Jensen BM, Anber K, Chen MM, Collins S, Esparza AN, Gieschen K, et al. Natural history of pressure injury among ethnically/racially diverse nursing home residents: the pressure ulcer detection study. J Gerontol Nurs. 2021;47(3):37–46. doi: 10.3928/00989134-20210210-03 33626163 PMC12951753

[25] Gefen A, Kolsi J, King T, Grainger S, Burns M. Modelling the cost-benefits arising from technology-aided early detection of pressure ulcers. Wounds Int. 2020;11(1):12–7.

[26] Scafide KN, Narayan MC, Arundel L. Bedside technologies to enhance the early detection of pressure injuries: a systematic review. J Wound Ostomy Continence Nurs. 2020;47(2):128–36. doi: 10.1097/WON.0000000000000626 32068647

[27] Oliveira AL, Moore Z, O Connor T, Patton D. Accuracy of ultrasound, thermography and subepidermal moisture in predicting pressure ulcers: a systematic review. J Wound Care. 2017;26(5):199–215. doi: 10.12968/jowc.2017.26.5.199 28475447

[28] Gefen A, Gershon S. An observational, prospective cohort pilot study to compare the use of subepidermal moisture measurements versus ultrasound and visual skin assessments for early detection of pressure injury. Ostomy Wound Manage. 2018;64(9):12–27. 30256748

[29] Charlton M, Stanley SA, Whitman Z, Wenn V, Coats TJ, Sims M, et al. The effect of constitutive pigmentation on the measured emissivity of human skin. PLoS One. 2020;15(11):e0241843. doi: 10.1371/journal.pone.0241843 33237918 PMC7688144

[30] Tian X, Fang L, Liu W. The influencing factors and an error correction method of the use of infrared thermography in human facial skin temperature. Build Environ. 2023;244:110736.

[31] Infrared camera accuracy and uncertainty in plain language. 2023 [Accessed 2025 April 29]. https://www.flir.com/discover/rd-science/infrared-camera-accuracy-and-uncertainty-in-plain-language/

[32] Aloweni FAB, Ang SY, Chang YY, Ng XP, Teo KY, Choh ACL. Evaluation of infrared technology to detect category I and suspected deep tissue injury in hospitalised patients. J Wound Care. 2019;28(Sup12):S9–16. 10.12968/jowc.2019.28.Sup12.S9 31825768

[33] Sudat SEK, Wesson P, Rhoads KF, Brown S, Aboelata N, Pressman AR. Racial disparities in pulse oximeter device inaccuracy and estimated clinical impact on COVID-19 treatment course. Am J Epidemiol. 2023;192(5):703–13. 36173743 10.1093/aje/kwac164 PMC9619495

[34] Norton HL. Variation in pulse oximetry readings: melanin, not ethnicity, is the appropriate variable to use when investigating bias. Anaesthesia. 2022;77(3):354–5. doi: 10.1111/anae.15620 34766336

[35] Bhavani SV, Wiley Z, Verhoef PA, Coopersmith CM, Ofotokun I. Racial differences in detection of fever using temporal vs oral temperature measurements in hospitalized patients. JAMA. 2022;328(9):885–6. 36066526 10.1001/jama.2022.12290 PMC9449792

[36] Amalu W. International academy of clinical thermology quality assurance guidelines. 2015 [Accessed 2024 June 25]. https://www.advancedthermalimagingllc.com/wp-content/uploads/2020/04/IACT-Standards-and-Guidelines.pdf

[37] Schwartz RMD. Guidelines for point of care medical thermography. 2024 [Accessed 2024 June 26]. https://aathermology.org/wp-content/uploads/2018/04/Guidelines-for-Point-of-Care-Medical-Thermography.pdf

[38] Monk E. Monk skin tone scale. 2022.

[39] Ly BCK, Dyer EB, Feig JL, Chien AL, Del Bino S. Research techniques made simple: cutaneous colorimetry: a reliable technique for objective skin color measurement. J Invest Dermatol. 2020 Jan;140(1):3–12 e1. 31864431 10.1016/j.jid.2019.11.003

[40] Andreassi L, Flori L. Practical applications of cutaneous colorimetry. Clin Dermatol. 1995;13(4):369–73. doi: 10.1016/0738-081x(95)00069-r 8665445

[41] Sonenblum SE, Patel R, Phrasavath S, Xu S, Bates-Jensen BM. Using technology to detect erythema across skin tones. Adv Skin Wound Care. 2023;36(10):524–33. 37729162 10.1097/ASW.0000000000000043 PMC10545068

[42] Bates-Jensen BM, Jordan K, Jewell W, Sonenblum SE. Thermal measurement of erythema across skin tones: Implications for clinical identification of early pressure injury. J Tissue Viability. 2024;33(4):745–52. doi: 10.1016/j.jtv.2024.08.002 39214728

[43] Dadzie OE, Sturm RA, Fajuyigbe D, Petit A, Jablonski NG. The Eumelanin Human Skin colour scale: a proof-of-concept study. Br J Dermatol. 2022;187(1):99–104. doi: 10.1111/bjd.21277 35349165

[44] WoundVision LLC. WoundVision 510(k) Summary. Indianapolis, IN; 2013.

[45] Jiang X, Wang Y, Wang Y, Zhou M, Huang P, Yang Y, et al. Application of an infrared thermography-based model to detect pressure injuries: a prospective cohort study. Br J Dermatol. 2022;187(4):571–9. doi: 10.1111/bjd.21665 35560229

[46] Sprigle S, Linden M, McKenna D, Davis K, Riordan B. Clinical skin temperature measurement to predict incipient pressure ulcers. Adv Skin Wound Care. 2001;14(3):133–7. doi: 10.1097/00129334-200105000-00010 11905978

[47] Judy D, Brooks B, Fennie K, Lyder C, Burton C. Improving the detection of pressure ulcers using the TMI ImageMed system. Adv Skin Wound Care. 2011;24(1):18–24. doi: 10.1097/01.ASW.0000392925.83594.50 21173587

