# Correction: Impact of skin tone, environmental, and technical factors on thermal imaging

**DOI:** 10.1371/journal.pone.0349788

**Published:** 2026-05-22

**Authors:** Sharon Eve Sonenblum, Kathleen Jordan, Glory Tomi John, Andrew Chung, Miriam Asare-Baiden, Jordan Pelkmans, Judy Wawira Gichoya, Vicki Stover Hertzberg, Joyce C. Ho

In Fig 3, the figure should not display the intended control region size and positioning. Please see the correct Fig 3 here.

In the Methods subsection of the Abstract, there is an error in the last sentence of the paragraph. The correct sentence is: The cooling/imaging procedure was repeated using the alternate camera, and data were analyzed using mixed-effects model.

In the Data processing subsection of the Materials and methods, there are errors in the first and third sentence of the third paragraph. The correct sentences are: A standardized control region was automatically created as an ellipse scaled proportionally to the cooling ROI, with an area measuring approximately 1/4th (25%) of the area of the cooling ROI. The control region was positioned superior along the x-axis of the image, with a gap of approximately one-eighth the ROI diameter between the edges of the two regions.

**Fig 3:**
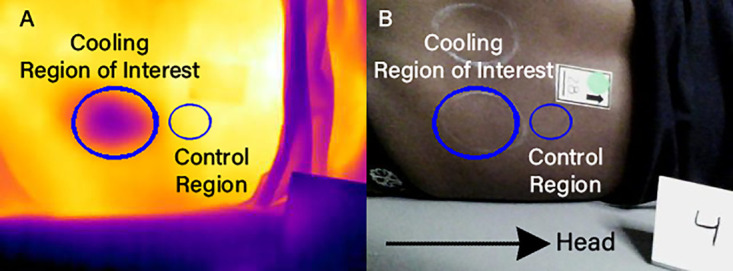
Regions of interest for the cooling protocol shown on (A) thermal and (B) optical images after alignment. The cooling ROI (large ellipse) is positioned over the posterior superior iliac spine (PSIS); the control ROI (small ellipse) is located superior to the PSIS.
