# Supplementary material for: Impact of skin tone, environmental, and technical factors on thermal imaging
Source: PLoS One. 2025 Sep 10;20(9):e0325091. doi: 10.1371/journal.pone.0325091 (PMC12422453; doi:10.1371/journal.pone.0325091)
Supplement: S1 File — (PDF) [file pone.0325091.s001.pdf]

Camera

Table 1. Average temperatures (°C, Mean ± SD) calculated using within-subject averages, separated by thermal camera (E8XT vs. ONE Pro), cooling condition (Baseline vs. Cooling), and region of interest (Cooling ROI vs. control region).

Cooling Condition	Baseline		Cooled		Cooled - Baseline	
**Camera**	**E8XT**	**ONE Pro**	**E8XT**	**ONE Pro**	**E8XT**	**ONE Pro**
Cooling Region of Interest	32.0 ± 1.4	29.6 ± 1.3	27.8 ± 1.4	27.3 ± 1.1	-4.2 ± 1.0	-2.3 ± 1.0
Control Region	32.2 ± 1.1	29.9 ± 1.1	32.0 ± 1.0	30.5 ± 1.0	-0.2 ± 0.8	0.6 ± 0.9
Cooling Region of Interest – Control Region	-0.3 ± 0.6	-0.3 ± 0.5	-4.2 ± 0.9	-3.2 ± 0.8		

Distance

Table 2. Average temperatures (°C, Mean ± SD) calculated using within-subject averages, separated by thermal camera (E8XT vs. ONE Pro), cooling condition (Base vs. Cool), skin to camera distance (35 cm vs. 50 cm), and region of interest (Cooling ROI vs. control region).

Cooling Condition	Baseline				Cooled				Cooled - Baseline			
**Camera**	**E8XT**		**ONE Pro**		**E8XT**		**ONE Pro**		**E8XT**		**ONE Pro**	
**Distance**	**35 cm**	**50 cm**	**35 cm**	**50 cm**	**35 cm**	**50 cm**	**35 cm**	**50 cm**	**35 cm**	**50 cm**	**35 cm**	**50 cm**
Cooling Region of Interest	32.0 ± 1.4	31.9 ± 1.3	29.6 ± 1.4	29.5 ± 1.3	27.8 ± 1.4	27.8 ± 1.4	27.2 ± 1.2	27.3 ± 1.1	-4.3 ± 1.1	-4.1 ± 1.0	-2.4 ± 1.1	-2.2 ± 1.0
Control Region	32.3 ± 1.2	32.2 ± 1.2	29.9 ± 1.2	29.8 ± 1.2	32.0 ± 1.1	31.9 ± 1.1	30.5 ± 1.1	30.5 ± 0.9	-0.2 ± 0.8	-0.3 ± 0.7	0.5 ± 1.1	0.7 ± 1.0
Cooling Region of Interest – Control Region	-0.2 ± 0.7	-0.3 ± 0.6	-0.3 ± 0.5	-0.3 ± 0.5	-4.3 ± 0.9	-4.1 ± 1.0	-3.3 ± 0.8	-3.2 ± 0.8				

Posture

Table 3. Average temperatures (°C, Mean ± SD) calculated using within-subject averages, separated by thermal camera (E8XT vs. ONE Pro), cooling condition (Baseline vs. Cooling), posture (knee back, knee forward, or knees stacked), and region of interest (Cooling ROI vs. control region).

Cooling Condition	Baseline						Cooled						Cooled - Baseline					
Camera	E8XT			ONE Pro			E8XT			ONE Pro			E8XT			ONE Pro		
Posture	Knee Back	Knee Forward	Knees Stacked	Knee Back	Knee Forward	Knees Stacked	Knee Back	Knee Forward	Knees Stacked	Knee Back	Knee Forward	Knees Stacked	Knee Back	Knee Forward	Knees Stacked	Knee Back	Knee Forward	Knees Stacked
Cooling Region of Interest	32.0 ± 1.4	32.0 ± 1.4	32.0 ± 1.3	29.4 ± 1.4	29.7 ± 1.3	29.6 ± 1.4	27.8 ± 1.4	27.9 ± 1.5	27.7 ± 1.4	27.1 ± 1.3	27.3 ± 1.2	27.3 ± 1.1	-4.2 ± 1.0	-4.1 ± 1.1	-4.3 ± 1.1	-2.3 ± 1.3	-2.4 ± 0.9	-2.3 ± 1.0
Control Region	32.2 ± 1.2	32.3 ± 1.2	32.3 ± 1.1	29.7 ± 1.3	30.1 ± 1.1	29.9 ± 1.2	32.0 ± 1.0	32.1 ± 1.1	31.9 ± 1.1	30.4 ± 1.2	30.5 ± 1.0	30.5 ± 0.9	-0.2 ± 0.8	-0.2 ± 0.9	-0.3 ± 0.8	0.7 ± 1.2	0.5 ± 1.0	0.6 ± 1.0
Cooling Region of Interest – Control Region	-0.2 ± 0.6	-0.3 ± 0.7	-0.3 ± 0.6	-0.3 ± 0.5	-0.4 ± 0.5	-0.3 ± 0.5	-4.2 ± 0.9	-4.2 ± 1.0	-4.2 ± 1.0	-3.3 ± 0.7	-3.2 ± 0.9	-3.2 ± 0.8						

Lighting

Table 4. Average temperatures (°C, Mean ± SD) calculated using within-subject averages, separated by thermal camera (E8XT vs. ONE Pro), cooling condition (Baseline vs. Cooling), lighting (ring light vs. room light), and region of interest (Cooling ROI vs. control region).

**Cooling Condition**	**Baseline**				**Cooled**				**Cooled - Baseline**			
**Camera**	**E8XT**		**ONE Pro**		**E8XT**		**ONE Pro**		**E8XT**		**ONE Pro**	
**Posture**	**Ring light**	**Room Light**	**Ring light**	**Room Light**	**Ring light**	**Room Light**	**Ring light**	**Room Light**	**Ring light**	**Room Light**	**Ring light**	**Room Light**
Cooling Region of Interest	32.0 ± 1.3	32.0 ± 1.4	29.5 ± 1.4	29.6 ± 1.3	27.8 ± 1.4	27.8 ± 1.4	27.3 ± 1.2	27.2 ± 1.1	-4.2 ± 1.1	-4.2 ± 1.0	-2.2 ± 1.0	-2.4 ± 1.0
Control Region	32.2 ± 1.1	32.2 ± 1.2	29.8 ± 1.2	30.0 ± 1.2	32.0 ± 1.1	32.0 ± 1.0	30.5 ± 1.1	30.5 ± 1.0	-0.3 ± 0.8	-0.2 ± 0.8	0.7 ± 1.0	0.5 ± 1.0
Cooling Region of Interest – Control Region	-0.2 ± 0.6	-0.3 ± 0.6	-0.3 ± 0.5	-0.4 ± 0.5	-4.2 ± 0.9	-4.2 ± 0.9	-3.2 ± 0.8	-3.2 ± 0.7				

Combined Model

Table 5. Linear-mixed effects model combining all factors gave comparable output to the independent models presented in the main text. Significance codes: *** p<0.001, ** p<0.01, * p<0.05, . p<0.1

Fixed Effects						
**Variable**	**Estimate**	**SE**	**df**	**t-value**	**p-value**	**Significance**
**Intercept**	-0.780	0.458	35.6	-1.702	0.097	.
**Cooling (vs. baseline)**	-2.801	0.156	1638.0	-17.992	<0.001	***
**Melanin Index**	0.005	0.008	35.0	0.617	0.542	
**Room lighting (vs. Ring Light)**	-0.020	0.036	1638.0	-0.548	0.584	
**50cm distance (vs. 35cm)**	0.062	0.036	1638.0	1.723	0.085	.
**Knee forward position**	-0.050	0.044	1638.0	-1.136	0.256	
**Knees stacked position**	-0.028	0.044	1638.0	-0.632	0.527	
**ONE Pro camera (vs. E8XT)**	0.445	0.036	1638.0	12.321	<0.001	***
**Cooling × Melanin Index**	-0.011	0.003	1638.0	-4.130	<0.001	***
**Random Effects**						
**Group**	**Variance**		**SD**			
**Subject ID (intercept)**	0.370		0.608			
**Residual**	0.548		0.740			

Model specification:
• Dependent variable: Cooling ROI – Control Region
• Random effect: Subject ID (intercept)
• Fixed effects: Baseline/Cooling condition, Melanin Index, Lighting, Distance, Body Position, Camera Type, and Baseline/Cooling Condition × Melanin Index Interaction

Model fit statistics:
• Observations: 1,680
• Subjects: 35
• REML criterion: 3920.2

Camera Specifications

Table 6. Technical specifications of the two different FLIR Thermal Cameras (FLIR Systems, Inc., Wilsonville, OR) used in the study

Camera	Thermal Image Resolution	Visual Image Resolution	Accuracy	Min Focus Distance	Frequency	Thermal sensitivity (N.E.T.D)	Temperature Range
E8XT	320 × 240 (76,800 pixels)	640 x 480	+/- 2°C or +/- 2% of reading	1.6 ft (0.5 m)	9 Hz	< 0.05°C (50mK)	-4°F to 1022°F (-20°C to 550°C)
ONE Pro	160 x 120 (19,2000 pixels)	1440 x 1080	±3°C or ±5%, typical Percent of the difference between ambient and scene temperature. Applicable 60 sec after start-up when the unit is within 15°C — 35°C and the scene is within 5°C — 120°C.	15 cm	8.7 Hz	70 mK	-20°C — 120°C (–4°F — 248°F) and 0°C — 400°C (32°F — 752°F)
